# Identification of the mitochondrial protein ADCK2 as a therapeutic oncotarget of NSCLC

**DOI:** 10.7150/ijbs.78354

**Published:** 2022-10-24

**Authors:** Jin-zhi Zhang, Jia Liu, Yi-xin Xu, Wang-yang Pu, Ming-jing Shen, Kan-qiu Jiang, Yi-ling Yang, Jingjing Lu, Zhengbo Deng, Yi Yang, Wei-hua Xu

**Affiliations:** 1 Department of Thoracic Surgery, the Second Affiliated Hospital of Soochow University, Suzhou, China.; 2 Department of Operation Room, the Second Affiliated Hospital of Soochow University, Suzhou, China.; 3 Changshu Hospital affiliated to Nanjing University of Chinese Medicine, Changshu, China.; 4 Department of Oncology, the Second Affiliated Hospital of Soochow University, Suzhou, China.; 5 Department of Radiotherapy and Oncology, Affiliated Kunshan Hospital of Jiangsu University, Kunshan, China.; 6 Department of Nuclear Medicine, the Affiliated Suzhou Science & Technology Town Hospital of Nanjing Medical University, Suzhou, China.

**Keywords:** NSCLC, ADCK2, mitochondrial function, therapeutic target, cancer growth

## Abstract

The aarF domain containing kinase 2 (ADCK2) is a mitochondria-locating protein, important for fatty acid metabolism and coenzyme Q biosynthesis. The bioinformatics results show that elevated* ADCK2* transcripts in NSCLC correlate with poor overall survival and poor anti-PD-1/PD-L1 therapy response. ADCK2 is overexpressed in local human NSCLC tissues and various primary and established NSCLC cells. In NSCLC cells, ADCK2 shRNA or CRISPR/Cas9 knockout remarkably suppressed cell viability, proliferation, cell cycle progression, cell mobility, and provoked cell apoptosis. Moreover, ADCK2 depletion disrupted mitochondrial functions in NSCLC cells, causing cytochrome C release, mitochondrial depolarization, DNA damage and ATP reduction. Contrarily, ectopic ADCK2 overexpression promoted NSCLC cell growth. Further studies revealed that ADCK2 depletion inactivated Akt-mTOR signaling in primary NSCLC cells. NSCLC xenograft growth in nude mice was significantly hindered after ADCK2 silencing or knockout. ADCK2 depletion, apoptosis induction and oxidative injury as well as ATP reduction and Akt-mTOR inactivation were detected in ADCK2-silenced or ADCK2-knockout NSCLC xenograft tissues. Together overexpressed ADCK2 is important for the growth of NSCLC cells, representing an important therapeutic molecular oncotarget.

## Introduction

Non-small cell lung cancer (NSCLC) represents the most prevalent lung cancer, and it accounts for 85-90% of all lung cancers 1, 2. Owing to the developments of early screening, emerging technologies and molecularly-targeted therapies, the prognosis for of NSCLC has been improved 3-7. It incidence is still rising in China and other Eastern countries 3-7. It is important to further research NSCLC tumorigenesis and progression's mechanisms 3-5, 8, 9.

Mitochondria are vital for ATP production and macromolecules biosynthesis in eukaryotic cells. Recent studies have also proposed that mitochondrial bioenergetics and signaling are essential for tumorigenesis and cancer progression 10, 11. Oxidative phosphorylation (OXPHOS) could be augmented in cancer cells of different background, including the primary and metastatic cancer cells 10-12. Increased OXPHOS and mitochondrial respiration can fuel cancer cells, required for cancer cell survival, proliferation and metastasis 10-12. Moreover, cancer cell mitochondrial OXPHOS dependency is associated with chemotherapy and radiotherapy resistance 10-12.

The aarF domain containing kinase 2 (ADCK2) locates at mitochondria with its function largely unknown. It is important for mitochondrial metabolism and bioenergetics 13. ADCK2 promotes the transport of lipids into mitochondria and it is essential for mitochondrial fatty acid β-oxidation and Coenzyme Q (CoQ) biosynthesis 13. Conversely, ADCK2 heterozygous (+/-) mice exhibited the impaired fatty acid oxidation and mitochondrial myopathy 13. ADCK2 silencing inhibited the viability of glioblastoma cells and estrogen receptor-positive breast cancer cells 14, 15. ADCK2 depletion suppressed tumor necrosis factor α (TNFα)-induced hypoxia-inducible factor-1 (HIF-1α) stability in cancer cells 16. However, there is no information available about ADCK2 in NSCLC. The results herein show that ADCK2 is overexpressed in NSCLC, which is required for growth of NSCLC cells.

## Materials and Methods

### Reagents

The anti-ADCK2 antibody was obtained from OriGene (Beijing, China). All other antibodies were provided by Cell Signaling Technologies (Beverly, MA, USA). The sequences and the associated viral constructs were all obtained from Genechem Co. (Shanghai, China). The fluorescence dyes, z-DEVD-cho and z-VAD-cho were from Biyuntian (Wuxi, China).

### Cells

A549 and NCI-H1944 cell lines, and BEAS-2 epithelial cells were described previously 17, 18. Primary NSCLC cells that were derived from human patients, pCan-1/-2/-3 and the primary human lung epithelial cells (pEpi) were reported before 17-19. All participants provided written-informed consent. Protocols were reviewed by the Ethics Committee of Soochow University, and conformed to the guidelines of Helsinki declaration.

### Human tissues

As described previously 18, twenty (n = 20) primary NSCLC patients (8 female and 12 male, all with written-informed consent, 42 to 71-year old, stage-III-VI), administered at the authors' institution, were enrolled. cancer tissues and the adjacent normal lung tissues were separated carefully. Tissue lysates were freshly prepared at the time of detection.

### ADCK2 shRNA or overexpression

The verified ADCK2 shRNA or the ADCK2 cDNA was cloned into a GV248 vector (from Li's group 20). The construct and the lentivirus package constructs were transfected to the HEK-293T cells, thereby generating shRNA lentiviral particles or ADCK2 overexpression lentiviral particles. The viral particles were then filtered and added (at MOI=20) to NSCLC/epithelial cells. Through puromycin stable cells were selected and ADCK2 silencing or overexpression were verified. The scramble control non-sense shRNA lentivirus (“shSCR”) or the empty vector (“Vec”) were stably transduced to control NSCLC/epithelial cells.

### ADCK2 KO

Using Lipofectamine 3000 (Invitrogen) protocol, a LentiCas9-puro construct, provided by Genechem, was transduced to cultured NSCLC cells for 36h, and stable cells were selected through selection by puromycin. A Lenti-CRISPR/Cas9-ADCK2-KO construct (with sgRNA targeting sequence: GACCCTGACAGACAAACGCC, and the PAM sequence, AGG) was synthesized by Genechem. The construct and lentivirus packaging and envelope constructs were transfected into HEK293T cells. The virus-containing medium was then collected after 24h and virus was filtered and was utilized to infect Cas9-expressing NSCLC cells. After 96h, cells were verified of *ADCK2* KO and single stable ADCK2 KO NSCLC cells were obtained.

### Other assays

NSCLC cells or the lung epithelial cells were seeded at optimal cell density and cultured for indicated time periods. CCK-8, colony formation assay, “Transwell” assays, nuclear EdU staining, cell immunofluorescence staining, and the single strand DNA (ssDNA) ELISA were described early 17, 18, 21-23. Detecting measuring mitochondrial membrane potential by JC-1, the caspase activity assays, Histone DNA ELISA, and TUNEL nuclear staining were reported early 17, 18. ATP contents in cellular and tissue lysates were measured as previously described 24. The mitochondrial complex I activity in the described NSCLC cells was analyzed by a commercial kit (Abcam, Shanghai, China) according to the attached protocols. Lipid peroxidation was tested by a thiobarbituric acid reactive substance (TBAR) kit (Cayman Chemical, MI) according to the attached protocol. Immunohistochemistry (IHC) protocols were reported early 25.The detailed protocols for the Western blotting and the qRT-PCR assays were described previously 17, 18, 21. The mRNA primer pair sequences were described previously 13. The un-cropped blotting images were presented in **Figure S1.**

### Constitutively-active mutant Akt1

The adenoviral constitutively-active Akt1 (caAkt1, S473D) construct, as reported 17, was utilized to transduce the primary NSCLC cells using the described protocols 17.

### Tumor xenograft study

All laboratory athymic nude mice (five-six week old, 18.3-19.2g weight, half male half female) were described early 21. NSCLC cells (at three million cells per mouse, in 120 μL basic medium-Matrigel mix) were injected subcutaneously into flanks of mice. Tumor volume and the animal body weights were measured, with the volume of each tumor detected as reported 26. Protocols were reviewed by the Institutional Animal Care & Use Committee (IACUC) and the Ethics Committee of Soochow University.

### Statistical analysis

Data were always presented as mean ± standard deviation. Statistical analyses were carried out as described 27. The *in vitro* experiments were always repeated five times. “n.s.” indicates non-statistical differences (***P*** > 0.05).

## Results

### Increased *ADCK2* transcripts in NSCLC correlates with poor overall survival and poor anti-PD-1/PD-L1 therapy response

**Figure 1:**
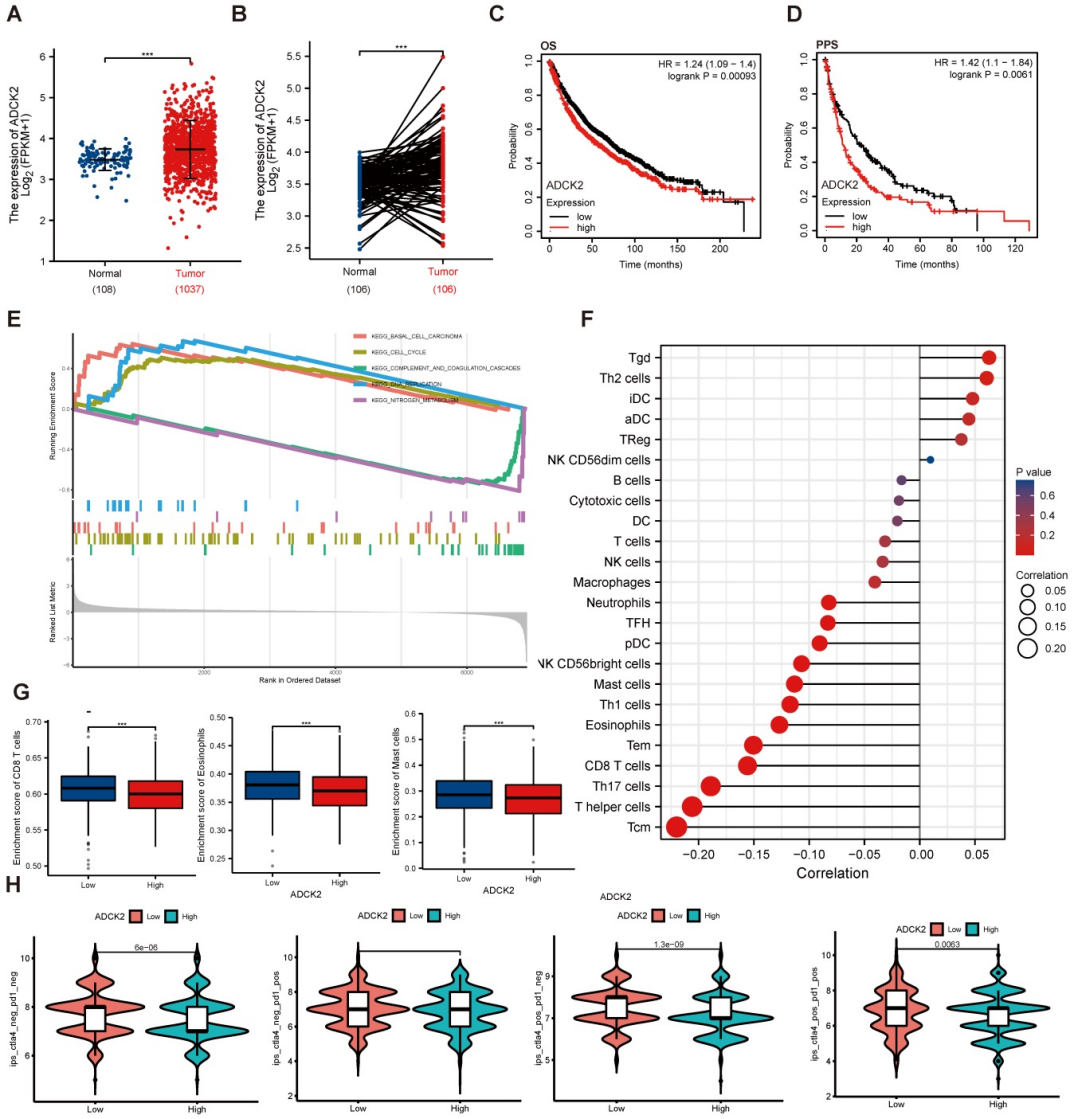
** Increased ADCK2 transcripts in NSCLC correlates with poor overall survival and poor anti-PD-1/PD-L1 therapy response.** TCGA cohorts revealed *ADCK2 mRNA* transcripts in the listed NSCLC tissues (“Tumor”) and normal lung tissues (“Normal”) (**A** and **B**). TCGA cohorts showed Kaplan Meier overall survival (OS) curve (**C**) and progression-free survival (PFS) curve (**D**) of *ADCK2*-low and *ADCK2*-high NSCLC patients (**B** and **C**). KEGG pathway analysis of *ADCK2*-associated DEGs and the enriched pathways (**E**). TCGA-LUAD/LUSC cohorts show the correlation between *ADCK* expression and immune cell infiltration (**F** and **G**). TCGA-LUAD/LUSC cohorts show predicted immunophenoscore (IPS) in *ADCK2*-low and *ADCK2*-highNSCLC patients (**H**). ***P < 0.001.

TCGA (The Cancer Genome Atlas)-LUAD/LUSC cohorts revealed that *ADCK2* mRNA transcript number in NSCLC tissues was higher when compared to that in lung epithelial tissues (Figure** 1A** and **B**). *ADCK2* mRNA overexpression in NSCLC was associated with poor overall survival (OS) (***P*** = 0.00093) and poor progression-free survival (PPS, ***P*** =0.0061) (Figure **1C**-**D**).

ADCK2 TCGA-LUAD/LUSC cohorts were further explored and differentially expressed genes (DEGs) were retrieved in NSCLC tissues. Gene Set Enrichment Analysis (GSEA) revealed a significant difference (false discovery rate < 5%, nominal ***P*** value < 25%) collection enrichment. KEGG pathway analysis further revealed that DEGs of basal cell carcinoma, cell cycle and DNA replication cascades were enriched in the *ADCK2*-high NSCLC (Figure **1E**). Whereas the gene set of complement and coagulation cascades as well as nitrogen metabolism showed enrichment in the *ADCK2*-low NSCLC (Figure **1E**).

Anti-PD-1/PD-L1 therapies are being applied for NSCLC patients 28, 29. We found that differences in ADCK2 expression are closely associated with immune cell infiltration in TCGA-LUAD/LUSC cohorts (Figure **1F**). Specifically, *ADCK2*-high expression is significantly correlated with less enrichment of CD8T cells, eosinophils and master cells (Figure **1G**). The immunophenoscore (IPS) measures the tumor immunogenicity. To access the potential relationship between ADCK2 expression and possible immunotherapy response, IPS were retrieved from TCGA-LUAD/LUSC. There are significant differences between the high ADCK2 gene expression group and the low ADCK2 gene expression group in all four immunotherapy regimens (Figure **1H**), and* ADCK2*-low predicts better anti-PD-1/PD-L1 therapy response (Figures **1H**). Together, the bioinformatics analysis results show that increased *ADCK2* transcripts in NSCLC correlates with poor overall survival and poor anti-PD-1/PD-L1 therapy response.

### ADCK2 is overexpressed in local NSCLC tissues

**Figure 2:**
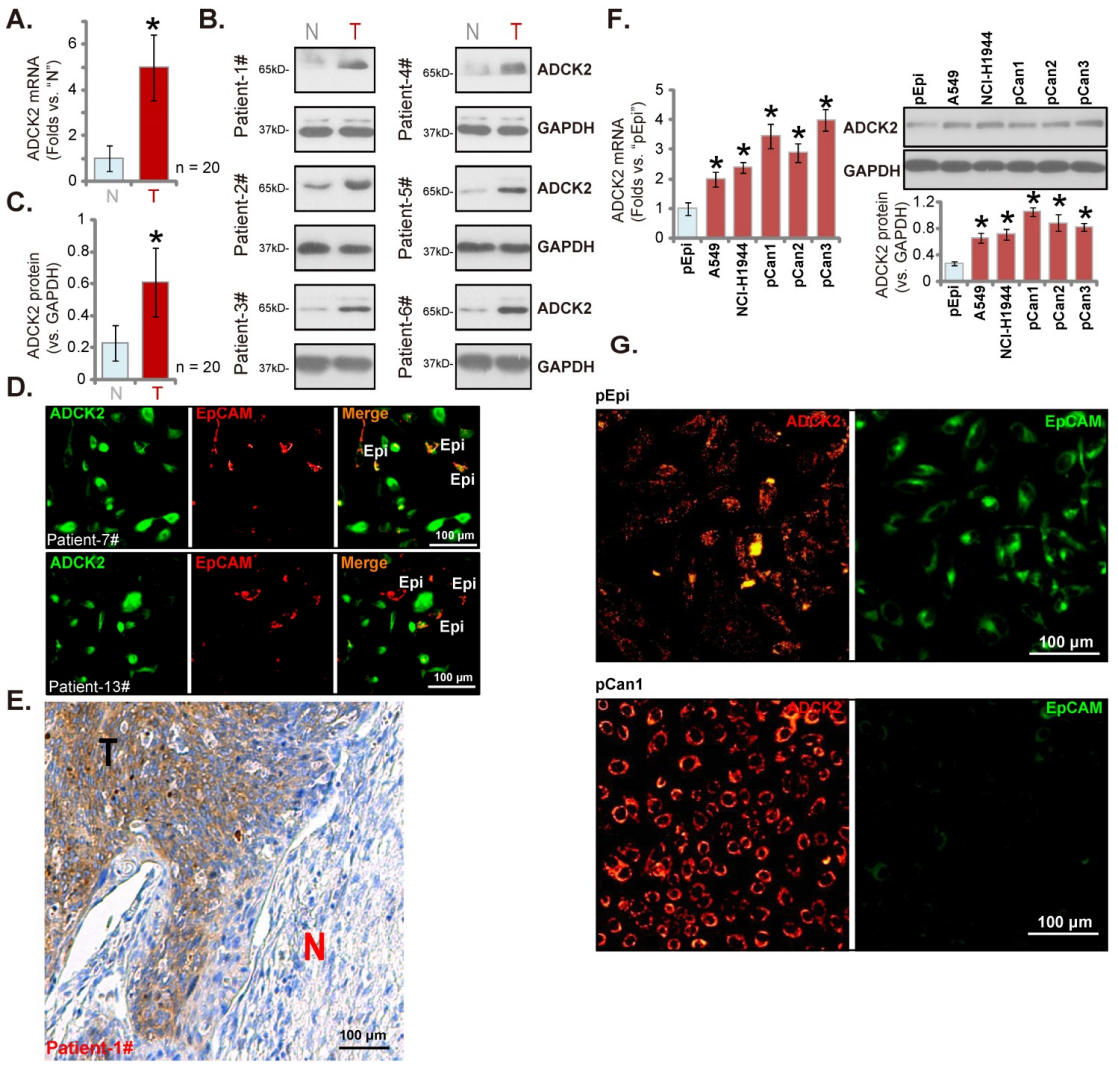
** ADCK2 is overexpressed in local NSCLC tissues.** *ADCK2* mRNA (**A**) and protein (**B** and **C**) expression in the described NSCLC tumor tissues (“T”) and the adjacent normal lung tissues (“N”) of 20 NSCLC patients was shown. The tissue immunofluorescence staining of ADCK2 (green fluorescence) and EpCAM (red fluorescence) at the junction of cancer and normal tissues of two NSCLC patients (Patient-7#/-13#) (**D**). The IHC images testing ADCK2 protein expression in NSCLC tumors (“T”) and normal lung tissues (“N”) of one representative NSCLC patients (Patient-1#) were shown (**E**). ADCK2 expression in the listed cells was shown (**F**). The immunofluorescence images of ADCK2 and EpCAM in the described cells were presented as well (**G**). “Epi” stands for epithelial cells (**D**). * ***P*** < 0.05 vs. “N” tissues or “pEpi” cells. Scale bar = 100 μm (**D**, **E** and **G**).

We next examined ADCK2 expression in local human NSCLC. Twenty (n = 20) primary human NSCLC patients with cancer resection surgery were enrolled. The fresh tumor tissues were obtained. In NSCLC tumor tissues (“T”) *ADCK2* mRNA expression was dramatically elevated when compared to its expression in the adjacent normal lung tissues (“N”) (Figure **2A**). ADCK2 protein was upregulated in six representative NSCLC tissues (tissues were derived from “Patient #1 to #6”) (Figure **2B**). After combining the 20 sets tissue specimen blotting data, results demonstrated that elevation of the ADCK2 protein in the NSCLC tissues was significant (Figure **2C**). The tissue immunofluorescence staining at the junction of cancer and normal tissues of two representative NSCLC patients (Patient-7#/-13#) showed that ADCK2 expression (green fluorescence) in cancer cells was higher than it in epithelial cells (“Epi”, EpCAM labeling, in red fluorescence) (Figure **2D**). The immunohistochemistry results in Figure **2E** confirmed ADCK2 overexpression in tumors (“T”) of the representative NSCLC patient (Patient-1#, see our previous studies 17, 18), whereas the expression was relatively low in cancer-surrounding normal lung tissues (“N”).

We next examined ADCK2 expression in different NSCLC cells, including A549/NCI-H1944 established cell lines and primary patient-derived NSCLC cells (namely “pCan-1”/“pCan-2”/“pCan-3”). *ADCK2* mRNA (results quantified in Figure **2F**) and protein (Figure **2F**) levels in the NSCLC cells were higher than those in primary human lung epithelial cells (“pEpi” 30, 31). Moreover, the immunofluorescence images in Figure **2G** further confirmed that ADCK2 expression (in red fluorescence) in primary NSCLC cells was higher than its expression in pEpi, the latter was labeled with the epithelial marker EpCAM (epithelial cell adhesion molecule, Alexa Fluor 488-conjugated, in green fluorescence). Therefore, ADCK2 is upregulated in local NSCLC tissues.

### ADCK2 depletion suppresses NSCLC cell survival, proliferation and cell motility

**Figure 3:**
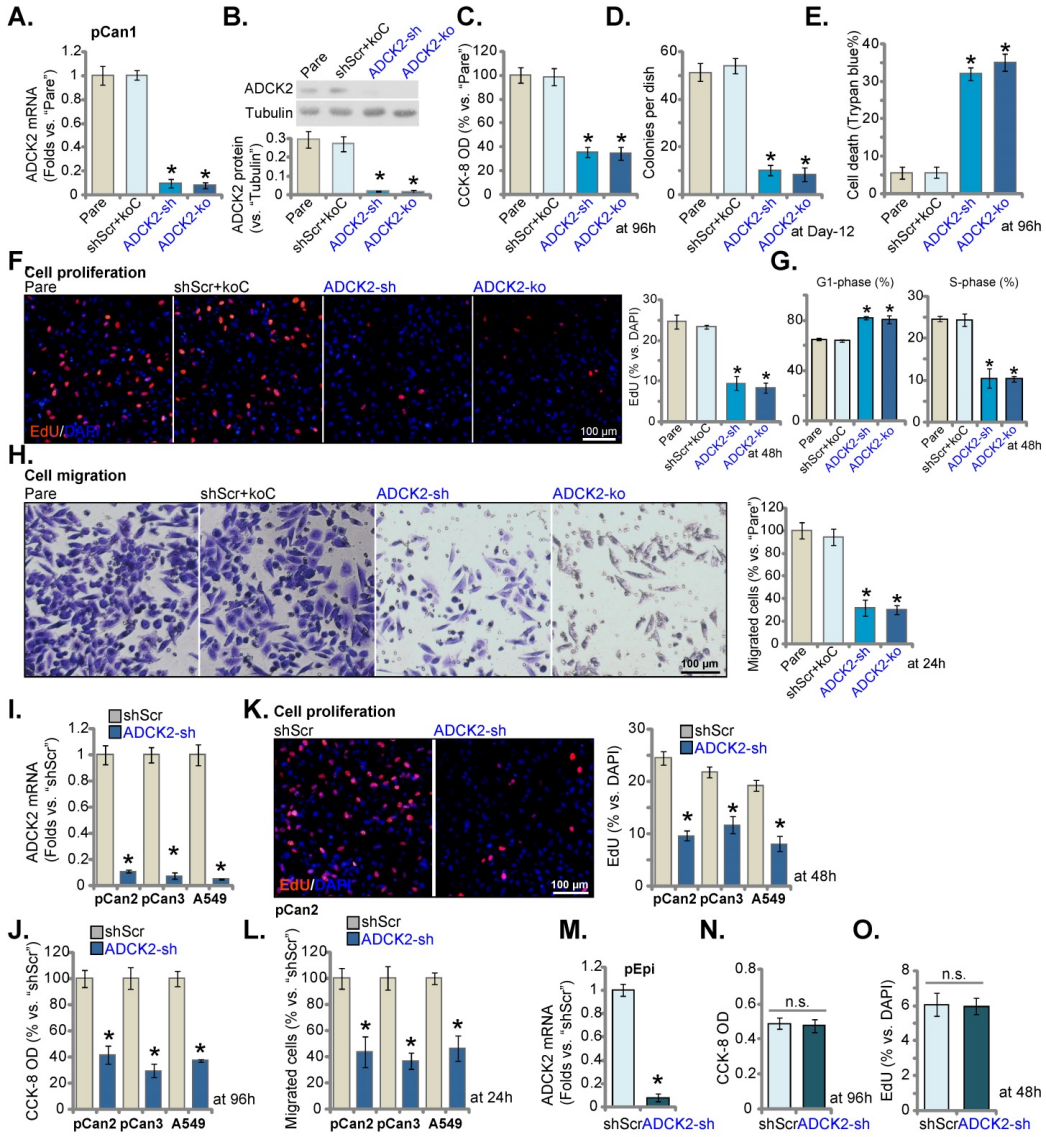
** ADCK2 depletion suppresses NSCLC cell survival, proliferation and cell motility.** Puromycin-selected pCan-1 cells, with the lentiviral ADCK2 shRNA (“ADCK2-sh”), the CRISPR/Cas9-ADCK2-KO construct (“ADCK2-ko”) or the lentiviral scramble non-sense control shRNA plus the CRISPR/Cas9 control empty construct (“shScr+koC”), were formed, expression of *ADCK2* mRNA (**A**) and protein (**B**) was shown; After culturing for designated time, cell viability (**C**), formed colonies (**D**), Trypan blue-positive staining cell ratio (**E**) and EdU-stained nuclei ratio (**F**), as well as cell cycle distribution (**G**) and *in vitro* cell migration (**H**) were tested. “pCan2” and “pCan3” primary cells, the established A549 cells, or “pEpi” lung epithelial cells, with “ADCK2-sh” or “shScr”, were established, and the expression of *ADCK2* mRNA was shown (**I** and **M**). After culturing for designated time, cell viability (**J** and **N**), EdU-stained nuclei ratio (**K** and **O**) and *in vitro* cell migration (**L**) were measured, with the results always quantified. “Pare” stands for parental control cells (same for all Figures). * ***P*** < 0.05 vs. “Pare” or “shScr”. Scale bar = 100 µm.

The lentiviral particles encoding the ADCK2 shRNA were transfected to the pCan-1 cells. The stable pCan-1 cells, “ADCK2-sh”, were established after selection. Also, a CRISPR/Cas9-ADCK2-KO construct was stably transduced to the Cas9-expressing pCan-1 cells to establish single stable “ADCK2-ko” cells. The lentiviral scramble control nonsense shRNA (“shScr”) and the CRISPR/Cas9 empty construct (“koC”) were tranduced to the control cells (“shScr+koC”). *ADCK2* mRNA decreased over 90% in ADCK2-sh and ADCK2-ko pCan-1 cells (Figure **3A**). ADCK2 protein expression was depleted as well (Figure **3B**). Figure **3C** demonstrated that ADCK2 silencing/KO decreased viability (CCK-8 OD) of the pCan-1 cells. Moreover, the pCan-1 cell colony number was significantly reduced in ADCK2-sh and ADCK2-ko pCan-1 cells (Figure **3D**). The increased percentage of Trypan blue-positive staining indicated that ADCK2 silencing or KO induced death of pCan-1 cells (Figure **3E**). These results implied that ADCK2 depletion led to potent cytotoxicity to pCan-1 cells.

Nuclear EdU staining assays were employed to test cell proliferation, and EdU-stained nuclei ratio (*vs.* DAPI) was significantly decreased in ADCK2-sh and ADCK2-ko pCan-1 cells (Figure **3F**), indicating proliferation inhibition by ADCK2 depletion. In addition, ADCK2 depletion in pCan-1 cells disrupted cell cycle progression, leading to G1-phase increasing but S-phase reduction (Figure **3G**). ADCK2 silencing/KO in pCan-1 cells potently inhibited cell migration (Figure **3H**). The shScr+koC treatment did not significantly alter ADCK2 expression (Figure **3A**-**B**) or pCan-1 cellular functions (Figure **3C**-**H**).

Two primary NSCLC cells, “pCan2” and “pCan3” 17, 18, and the immortalized A549 cells were cultured and were stably transduced with ADCK2 shRNA lentiviral particles: “ADCK2-sh” cells. The control cells were stably transduced with “shScr”. *ADCK2* mRNA was silenced in the tested NSCLC cells with ADCK2 shRNA (Figure **3I**). In the tested NSCLC cells, ADCK2 shRNA reduced CCK-8 viability (Figure **3J**), decreased EdU-stained nuclei ratio , (Figure **3K**) and slowed *in vitro* cell migration (Figure **3L**).

In the pEpi primary lung epithelial cells, ADCK2 silencing by the same lentiviral shRNA (“ADCK2-sh”, Figure **3M**) was unable to significantly reduce CCK-8 viability (Figure **3N**) and EdU incorporation (Figure **3O**), supporting a cancer cell-specific activity by ADCK2 depletion.

### ADCK2 depletion activates apoptosis in NSCLC cells

**Figure 4:**
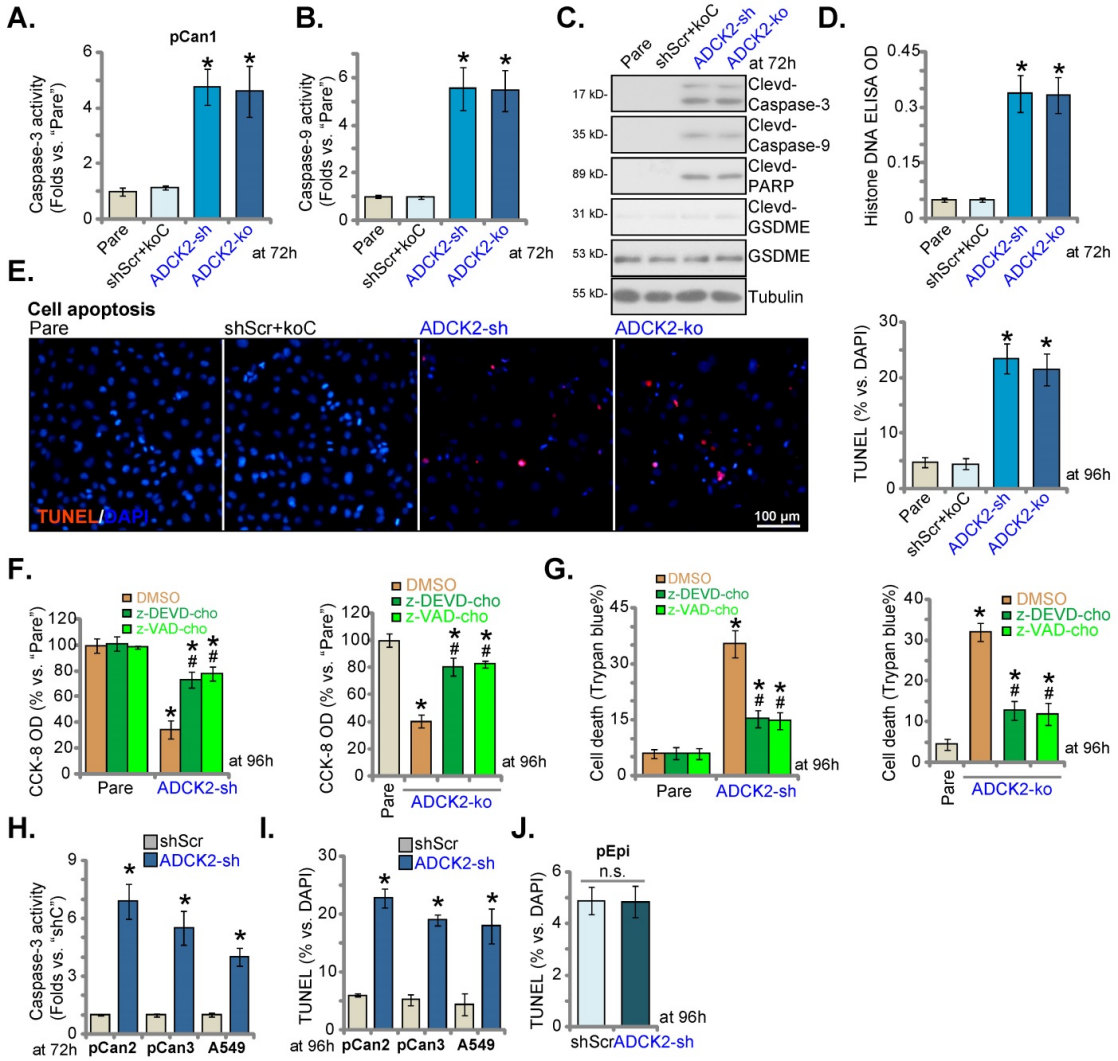
** ADCK2 depletion activates apoptosis in NSCLC cells.** “ADCK2-sh”, “ADCK2-ko” or “shScr+koC” pCan-1 cells were cultured for applied time periods; The relative activities of caspase-3 (**A**) and caspase-9 (**B**), apoptosis-related proteins expression (**C**) and Histone DNA contents (**D**) were tested. Cell apoptosis was examined by calculating TUNEL-stained nuclei ratio (**E**). The stable pCan-1 primary NSCLC cells, co-treated with z-DEVD-cho (40 μM), z-VAD-cho (40 μM) or the vehicle control (0.1% DMSO), were stably infected with ADCK2-sh or ADCK2-ko, after 96h cell viability and death were respectively tested by CCK-8 (**F**) and Trypan blue staining (**G**) assays. “pCan2” and “pCan3” primary cells, A549 cells, or “pEpi” lung epithelial cells, with “ADCK2-sh” or “shScr”, were established and were cultured. The caspase-3 activity (**H**) and nuclear TUNEL percentage (**I** and **J**) were measured. * ***P*** < 0.05 vs. “Pare”/“shSCR” cells. **^#^ *P*** < 0.05 vs. “DMSO” group (**F** and **G**). Scale bar = 100 µm.

ADCK2 shRNA or KO induced cytotoxicity in NSCLC cells, we thus analyzed whether apoptosis was induced. Caspase-3 and caspase-9 activities were dramatically augmented in ADCK2-sh and ADCK2-ko pCan-1 cells (Figure **4A** and **B**). Further supporting caspase activation, we showed cleavages of caspase-3/-9 and PARP were robustly induced in pCan-1 cells with ADCK2 shRNA/KO (Figure **4C**), where the Histone-bound DNA contents were augmented (Figure **4D**). Expression of total and cleaved GSDME (Gasdermin E), the indicator of pyroptosis activation 32, was unchanged in ADCK2-silenced or ADCK2-depleted cells (Figure **4C**).

Confirming apoptosis induction, the ratio of TUNEL-positively stained nuclei was increased in pCan-1 cells after ADCK2 silencing or KO (Figure **4E**). shScr+koC treatment was unable to stimulate caspase-apoptosis activation in pCan-1 cells (Figure **4A**-**E**). Significantly, ADCK2-sh- and ADCK2-ko-caused viability decrease (Figure **4F**) and cell death (Figure **4G**) were ameliorated by the caspase inhibitors z-DEVD-cho and z-VAD-cho. Therefore, apoptosis induction should be the primary cause of ADCK2 silencing-caused NSCLC cell death.

In primary NSCLC cells (“pCan2” and “pCan3”) and A549 cells, ADCK2 silencing, by ADCK2-sh, significantly augmented the activity of caspase-3 (Figure **4H**) and increased the ratio of TUNEL-stained nuclei (Figure **4I**), supporting apoptosis induction. Interestingly, in normal “pEpi” cells, ADCK2-sh treatment failed to significantly increase nuclear TUNEL ratio/apoptosis (Figure **4J**), again indicating the cancer cell specific effect by ADCK2 depletion.

### ADCK2 depletion disrupts mitochondrial functions in NSCLC cells

**Figure 5:**
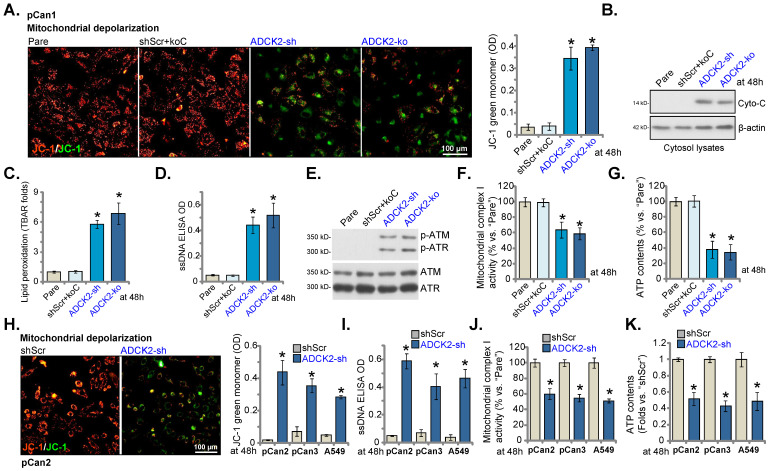
** ADCK2 depletion disrupts mitochondrial functions in NSCLC cells.** “ADCK2-sh”, “ADCK2-ko” or “shScr+koC” pCan-1 cells were cultured, mitochondrial depolarization (by recording intensity of JC-1 green monomers, **A**), cytosol cytochrome C protein levels (**B**), lipid peroxidation (TBAR intensity, **C**), as well as ssDNA contents (**D**), expression of DNA-damage related-proteins (**E**), mitochondrial complex I activity (**F**) and ATP contents (**G**) were tested. “pCan2” and “pCan3” primary cells or the established A549 cells, with “ADCK2-sh” or “shScr”, were cultured, mitochondrial depolarization (**H**), the ssDNA contents (**I**), mitochondrial complex I activity (**J**) and ATP contents (**K**) were tested similarly. * ***P*** < 0.05 vs. “Pare” or “shSCR”. Scale bar = 100 µm.

ADCK2 is a mitochondrial protein with the aarF domain, we therefore tested whether ADCK2 knockdown/depletion could disrupt normal functions of mitochondria. As shown in Figure **5A**, in pCan-1 NSCLC cells ADCK2 shRNA or KO provoked mitochondrial depolarization and induced accumulation of green JC-1 monomers. Cytochrome C was released from mitochondria, as cytochrome C levels in cytosol increased significantly in pCan-1 NSCLC cells with ADCK2 silencing/KO (Figure **5B**). Moreover, the increased lipid peroxidation, or TBAR activity enhancement, was observed (Figure **5C**). The ssDNA contents were elevated in ADCK2-sh and ADCK2-ko pCan-1 cells, indicating augmented DNA breaks (Figure **5D**). Moreover, ATM and ATR phosphorylation was significantly increased in ADCK2-depleted pCan-1 NSCLC cells (Figure **5E**). The mitochondrial complex I activity was significantly decreased in ADCK1-silenced/depleted cells (Figure **5F**). ATP contents were reduced as well in ADCK2-depleted pCan-1 cells (Figure **5G**). shScr+koC treatment did not significantly alter mitochondrial functions in pCan-1 cells (Figure **5A**-**G**).

In primary “pCan2” and “pCan3” cells and established A549 cells, ADCK2 shRNA similarly resulted in JC-1 green monomer increasing and mitochondrial depolarization (Figure **5H**), and augmented DNA breaks (Figure **5I**), but reduced mitochondrial complex I activity (Figure **5J**) and decreased ATP contents (Figure **5K**). These results implied that ADCK2 depletion in NSCLC cells disrupted normal functions of mitochondria.

### Ectopic overexpression of ADCK2 exerts pro-cancerous activity in NSCLC cells

**Figure 6:**
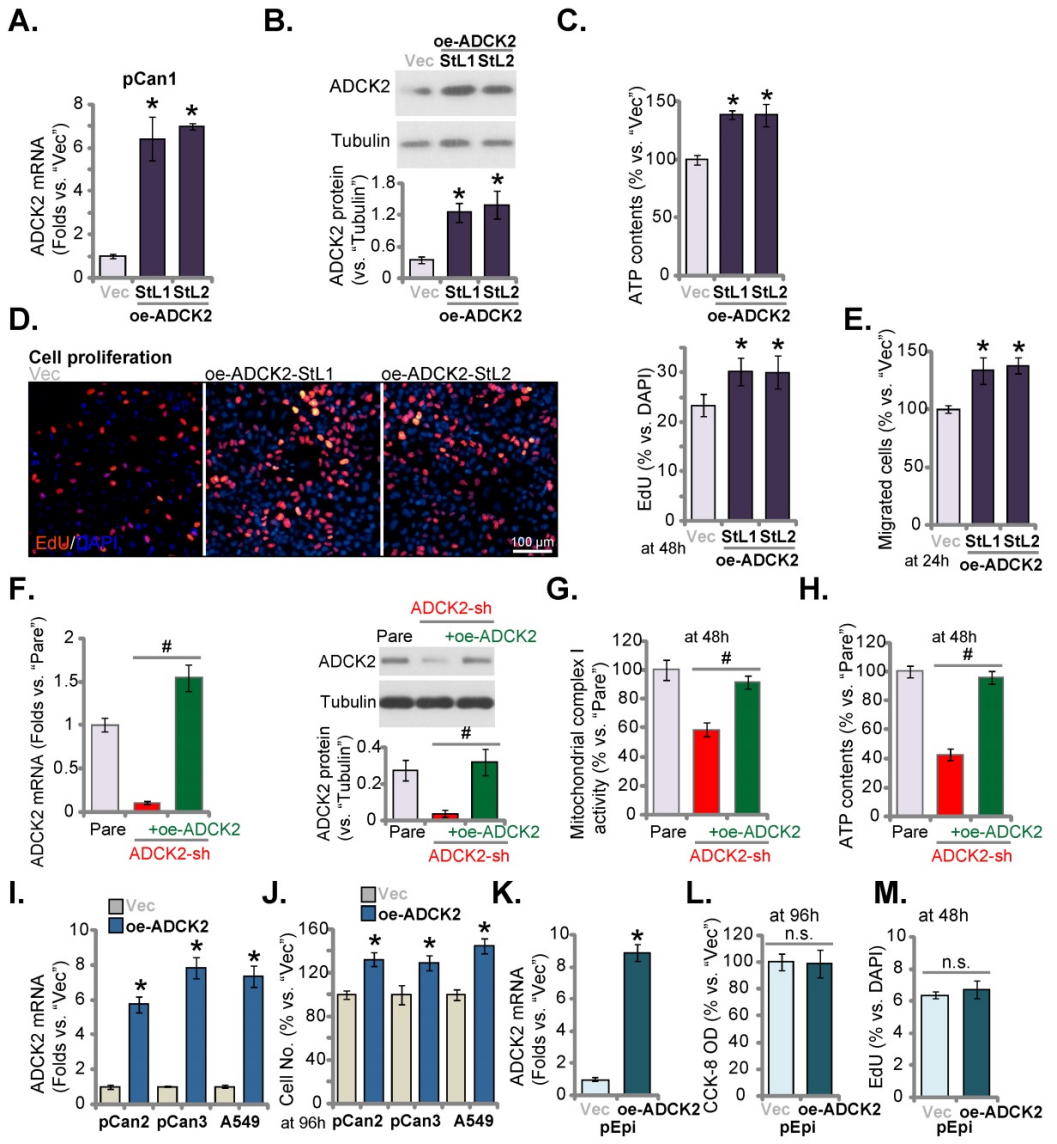
** Ectopic overexpression of ADCK2 exerts pro-cancerous activity in NSCLC cells.** Stable “pCan-1” cells, with the ADCK2-expressing lentiviral construct (oe-ADCK2-StL1 and oe-ADCK2-StL2, two stable selections) or the GV248 empty vector (“Vec”), were established, expression of *ADCK2* mRNA (**A**) and protein (**B**) was shown; After culturing for designated time, ATP contents (**C**), EdU-stained nuclei ratio (**D**), and migrated cell number (**E**) were measured and recorded. The pCan-1 primary NSCLC cells were infected with ADCK2 shRNA lentivirus (“ADCK2-sh”), or together with the ADCK2-expressing construct lentivirus (“+oe-ADCK2”), stable cells were formed, and ADCK2 expression was tested (**F** and **G**). After culturing for indicated time, mitochondrial complex I activity (**H**) and ATP contents (**I**) were measured. “pCan2” and “pCan3” primary cells, A549 cells, or “pEpi” lung epithelial cells with “oe-ADCK2” or “Vec” were established, expression of *ADCK2* mRNA was shown (**I** and **K**); The viable cell number was recorded after 96h (**J**); Cell viability (**L**) and proliferation (**M**) of pEpi cells were measured. * ***P*** < 0.05 vs. “Vec” cells. Scale bar = 100 µm.

Next lentiviral particles with ADCK2-expressing construct were added to pCan-1 cells and stable cell selections, namely oe-ADCK2-StL1 and oe-ADCK2-StL2, were formed through selection. *ADCK2* mRNA/protein expression was dramatically increased in oe-ADCK2 pCan-1 cells (Figure **6A** and **B**). As demonstrated, in oe-ADCK2 pCan-1 cells, the cellular ATP contents were significantly increased (Figure **6C**). Ectopic ADCK2 overexpression promoted pCan-1 proliferation, and the ratio of the EdU-stained nuclei was increased (Figure **6D**). Moreover, pCan-1 *in vitro* cell migration was enhanced as well after ADCK2 overexpression (Figure **6E**).

The rescue experiments were carried out. pCan-1 cells were stably infected with ADCK2 shRNA lentivirus (“ADCK2-sh”) or together with the ADCK2-expressing construct lentivirus (“+oe-ADCK2”). As shown, oe-ADCK2 restored *ADCK2* mRNA and protein expression in ADCK2-sh pCan-1 cells (Figure **6F**). Significantly, ADCK2-sh-induced mitochondrial complex I activity inhibition (Figure **6G**) and ATP reduction (Figure **6H**) were reversed by oe-ADCK2.

In other primary NSCLC cells (“pCan2” and “pCan3”) and established A549 cells, stable transfection of the ADCK2-expressing lentiviral construct (“oe-ADCK2”) similarly increased *ADCK2* mRNA expression (Figure **6I**). Consequently, the number of viable NSCLC cells was dramatically increased (Figure **6J**). Interestingly, in “pEpi” normal cells, ADCK2 overexpression using the same construct (“oeADCK2”, Figure **6K**) was unable to alter CCK-8 viability (Figure **6L**) and cell proliferation (Figure **6M**). These results again supported the selected effect of ADCK2 in cancerous cells.

### ADCK2 depletion inactivates Akt- mTOR signaling in primary NSCLC cells

**Figure 7:**
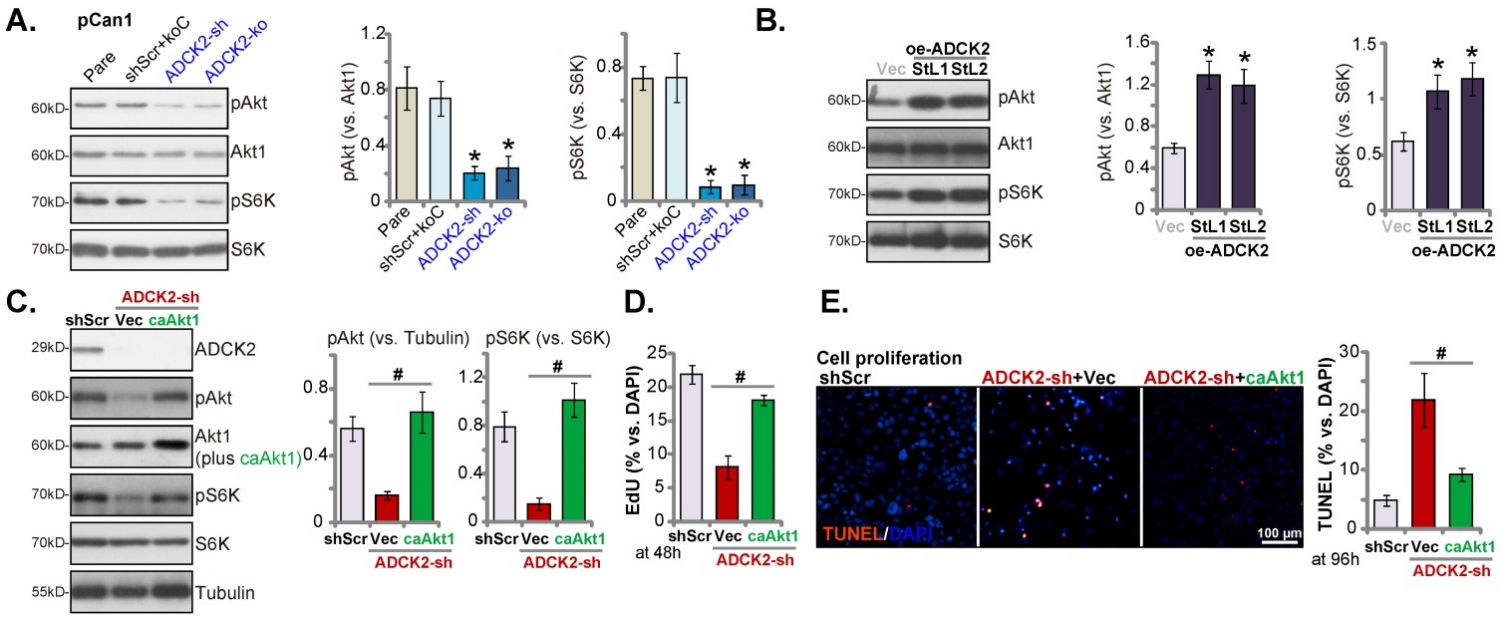
** ADCK2 depletion inactivates Akt-mTOR signaling in primary NSCLC cells.** Expression of listed proteins in “ADCK2-sh”, “ADCK2-ko” or “shScr+koC” pCan-1 cells, in oe-ADCK2-StL1 and oe-ADCK2-StL2 pCan-1 cells or in “Vec” pCan-1 cells was shown (**A** and **B**). ADCK2-sh pCan-1 cells were further stably transduced with a constitutively-active Akt1 (S473D, “caAkt1”) construct, and listed proteins were tested (**C**); After culturing for indicated time, EdU-stained nuclei ratio (**D**) and TUNEL-stained nuclei ratio (**E**) were measured. * ***P*** < 0.05 vs. “Pare” or “Vec”. **^#^ *P*** < 0.05 (**D** and **E**). Scale bar = 100 µm.

Considering that ATP production and energy are vital for activation of Akt-mTOR signaling 33-35, the vital oncogenic cascade essential for NSCLC development and progression 36-39. In pCan-1 cells, ADCK2 silencing or depletion resulted in robust inhibition of phosphorylation Akt (Ser-473, the same below) and S6K1 (Figure **7A**), suggesting that ADCK2 depletion indeed hindered Akt-mTOR activation in primary NSCLC cells. Conversely, ADCK2 overexpression further enhanced Akt-mTOR activation in pCan-1 cells (Figure **7B**), as phosphorylation Akt and S6K1 was augmented in oe-ADCK2-StL1 and oe-ADCK2-StL2 pCan-1 cells (Figure **7B**).

To explore the relationship between ADCK2 depletion-induced anti-NSCLC cell activity and Akt-mTOR inactivation, a constitutively-active Akt1 (S473D, “caAkt1” 17) was transduced to ADCK2 shRNA-expressing pCan-1 cells. As demonstrated, caAkt1 was able to completely restore Akt-S6K phosphorylation in shADCK2-expressing pCan-1 cells (Figure **7C**), but did not bring back ADCK2 protein expression (Figure **7C**). Significantly, shADCK2-caused anti-proliferative activity (Figure **7D**) and apoptosis (Figure **7E**) were ameliorated by caAkt1. Thus Akt-mTOR inactivation should be one primary mechanism of ADCK2 depletion-induced activity against NSCLC cells.

### ADCK2 depletion slows NSCLC xenograft growth in nude mice

**Figure 8:**
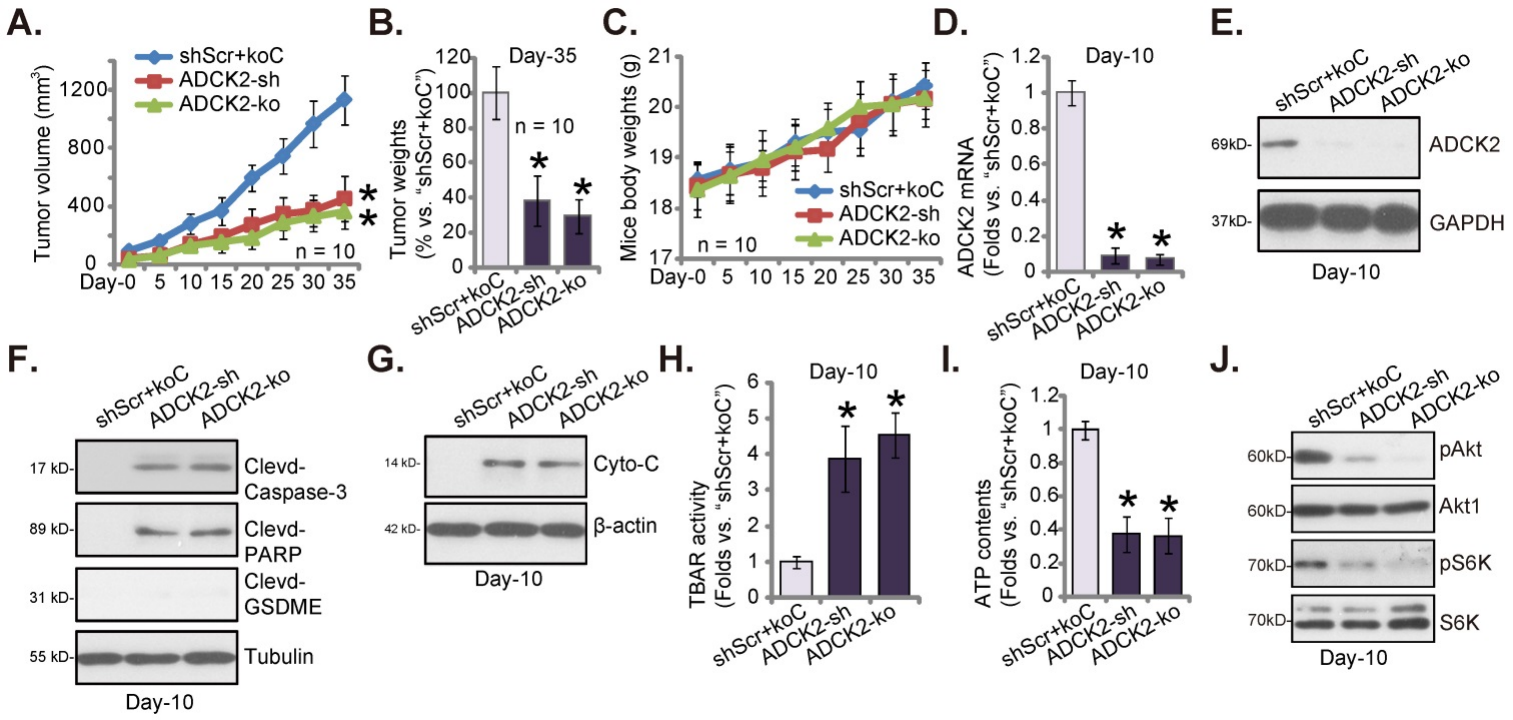
** ADCK2 depletion slows NSCLC xenograft growth in nude mice.** “ADCK2-sh”, “ADCK2-ko” or “shScr+koC” pCan-1 cells were *s.c.* injected to the flanks of nude mice. Within two weeks of cell implantation, NSCLC xenograft tumors were established (“Day-0”), and tumor volumes (**A**) and mice body weights (**C**) were recorded. At Day-35, xenografts were isolated and weighted (**B**). At Day-10, one xenograft tumor per group was isolated, in tumor lysates expression of listed genes and proteins was tested (**D**-**G** and** J**), with TBAR activity (**H**) and ATP contents (**I**) measured as well. * ***P*** < 0.05 vs. “shScr+koC” group.

Lastly, ADCK2-silenced pCan-1 cells (“ADCK2-sh”), the ADCK2 KO pCan-1 cells (“ADCK2-ko”) as well as the control “shScr+koC” pCan-1 cells were injected* s.c.* to nude mice's flanks. Two weeks later, NSCLC xenografts were formed (labeled as “Day-0”). Figure **8A** demonstrated that the growth of the ADCK2-silenced pCan-1 xenografts and the ADCK2 KO pCan-1 xenografts was dramatically slower than that of shScr+koC NSCLC xenografts. At Day-35, NSCLC xenografts were isolated very carefully and individually weighted. pCan-1 xenografts with ADCK2 silencing or KO were dramatically lighter than shScr+koC xenografts (Figure **8B**). The mice body weights showed no significant difference among the three experimental groups (Figure **8C**).

At experimental Day-10, one xenograft per group was separated and analyzed. *ADCK2* mRNA/protein expression was decreased significantly in the ADCK2-sh-xenograft tumors and the ADCK2-ko-xenograft tumors (Figure **8D** and **E**). Notably, caspase-3 and PARP cleavages were significantly augmented in ADCK2-depleted tumors (Figure **8F**), indicating apoptosis induction. The cleaved-GSDME expression was unchanged (Figure **8F**). In addition, levels of the cytosol cytochrome C were increased in NSCLC xenograft tumors bearing ADCK2 shRNA or ADCK2 KO construct (Figure **8G**). Moreover, the increased TBAR activity in ADCK2-sh- and ADCK2-ko-xenograft tumor tissues implied oxidative injury and mitochondrial injury (Figure **8H**). Further studies showed that ATP contents were decreased after ADCK2 silencing or KO in pCan-1 xenograft tissues (Figure **8I**). Moreover, phosphorylation of Akt-S6K1 were remarkably decreased in ADCK2-depleted tumors (Figure **8J**). Thus, ADCK2 depletion decreased ATP contents and inhibited Akt-mTOR activation in pCan-1 xenografts.

## Discussion

The prognosis of the recurrent, metastatic, therapy-resistant and other advanced NSCLC is still poor. The five-year survival has not been significantly improved recently 40, 41. It emphasizes the urgent need for identifying novel therapeutic targets with high efficiency for NSCLC diagnosis and treatment 37, 42-44. ADCK2 is predicted as an atypical mitochondrial kinase that is vital for fatty acid metabolism and CoQ biosynthesis 13. Here the bioinformatics results show that elevated* ADCK2* transcripts in NSCLC correlate with poor overall survival and poor anti-PD-1/PD-L1 therapy response. Moreover, ADCK2 overexpression was detected in local NSCLC tissues and different NSCLC cells. While its expression in lung epithelial tissues and cells is relatively low.

Few studies have reported ADCK2's potential function in cancer cells. A functional viability profile study in breast cancer has identified ADCK2 as a key gene for breast cancer cell survival 15. ADCK2 silencing potently inhibited ER-positive breast cancer cell survival 15. ADCK2 is important for estrogen-induced breast cancer cell progression 15. ADCK2 silencing inhibited estrogen signaling and decreased expression of ER target genes 15. Conversely, estrogen stimulation increased ADCK2 expression in breast cancer cells, while its expression was decreased after treatment with the ER inhibitors 15. More importantly, ADCK2 co-immunoprecipitated with ERα in breast cancer cells 15. A functional genomics screen study has shown that TNFα-induced ADCK2 expression in human osteosarcoma cells and prostate cancer cells inhibited ROS production, and it was required for HIF-1α stability 16. Conversely, depletion of ADCK2 largely inhibited TNFα-induced HIF-1α stability in cancer cells 16.

The work herein supported that ADCK2 exerted significant pro-cancerous activity. In NSCLC cells, ADCK2 shRNA or KO robustly suppressed malignant behaviors, and provoked cell apoptosis. Contrarily, ectopic ADCK2 overexpression augmented NSCLC cell growth and accelerated *in vitro* cell migration. The growth of NSCLC xenografts in nude mice was significantly hindered after ADCK2 knockdown or KO.

NSCLC, like other common tumors, exhibits elevated mitochondrial metabolism, including glycolysis and glucose oxidation 10-12. The biosynthesis and update of heme were significantly augmented in NSCLC cells, correlating with upregulation of ALAS1 and SLC48A1 45. ALAS1 or SLC48A1 overexpression promoted oxygen consumption and ATP generation, associated with the enhanced tumorigenic potential of NSCLC 45. Conversely, cyclopamine tartrate (CycT), by inhibiting heme biosynthesis, remarkably hindered NSCLC cell growth 46.

ADCK2 is required for lipid transport into mitochondria, and following fatty acid β-oxidation and CoQ biosynthesis 13. Here we found that ADCK2 is important for maintaining normal mitochondrial functions in NSCLC cells. ADCK2 depletion disrupted mitochondrial functions in NSCLC cells, causing cytochrome C release, mitochondrial depolarization and ATP depletion. Moreover, increased levels of cytosol cytochrome C and lipid peroxidation were detected in ADCK2-sh- and ADCK2-ko-NSCLC xenograft tumor tissues. Based on these results, we proposed that ADCK2 was required for mitochondrial metabolism in NSCLC cells and depletion of ADCK2 resulted in mitochondrial dysfunction, thereby arresting cancer cell growth. The underlying mechanisms for ADCK2-driven NSCLC cell growth require further characterizations.

Akt-mTOR aberrant activation contributes significantly to tumorigenesis and cancer progression in NSCLC 36, 39. We have previously shown that a highly-potent mTOR kinase inhibitor PQR620 arrested growth of NSCLC cells 17. Here we found that ADCK2 shRNA or KO inactivated Akt-mTOR signaling in NSCLC cells. Akt-mTOR inhibition was detected as well in ADCK2-depleted NSCLC xenograft tissues. While caAkt1 recovered Akt-mTOR activation and alleviated ADCK2 silencing-induced anti-NSCLC cell activity. Therefore, ADCK2-promoted NSCLC cell progression is mediated by promoting activation of the Akt-mTOR cascade.

## Conclusion

Overexpressed ADCK2 is important for the growth of NSCLC cells, representing an important therapeutic molecular oncotarget.

## Supplementary Material

Supplementary figure.

## References

[1] Siegel RL, Miller KD, Jemal A (2020). Cancer statistics, 2020. CA: a cancer journal for clinicians.

[2] Siegel RL, Miller KD, Jemal A (2019). Cancer statistics, 2019. CA: a cancer journal for clinicians.

[3] Rosell R, Karachaliou N (2015). Lung cancer in 2014: optimizing lung cancer treatment approaches. Nature reviews Clinical oncology.

[4] Neal JW, Gainor JF, Shaw AT (2015). Developing biomarker-specific end points in lung cancer clinical trials. Nature reviews Clinical oncology.

[5] Keith RL, Miller YE (2013). Lung cancer chemoprevention: current status and future prospects. Nature reviews Clinical oncology.

[6] Cortes-Dericks L, Galetta D (2019). The therapeutic potential of mesenchymal stem cells in lung cancer: benefits, risks and challenges. Cell Oncol (Dordr).

[7] Huang J, Li Y, Lu Z, Che Y, Sun S, Mao S (2019). Analysis of functional hub genes identifies CDC45 as an oncogene in non-small cell lung cancer - a short report. Cell Oncol (Dordr).

[8] He Y, Chen L, Zhao L, Dang S, Liu G, Sasada S (2021). Genomic and transcriptional alterations in first-line chemotherapy exert a potentially unfavorable influence on subsequent immunotherapy in NSCLC. Theranostics.

[9] Wang D, Zhao C, Xu F, Zhang A, Jin M, Zhang K (2021). Cisplatin-resistant NSCLC cells induced by hypoxia transmit resistance to sensitive cells through exosomal PKM2. Theranostics.

[10] Burke PJ (2017). Mitochondria, Bioenergetics and Apoptosis in Cancer. Trends in cancer.

[11] Porporato PE, Filigheddu N, Bravo-San Pedro JM, Kroemer G, Galluzzi L (2018). Mitochondrial metabolism and cancer. Cell Res.

[12] Zong WX, Rabinowitz JD, White E (2016). Mitochondria and Cancer. Molecular cell.

[13] Vazquez-Fonseca L, Schafer J, Navas-Enamorado I, Santos-Ocana C, Hernandez-Camacho JD, Guerra I (2019). ADCK2 Haploinsufficiency Reduces Mitochondrial Lipid Oxidation and Causes Myopathy Associated with CoQ Deficiency. Journal of clinical medicine.

[14] Wiedemeyer WR, Dunn IF, Quayle SN, Zhang JH, Chheda MG, Dunn GP (2010). Pattern of retinoblastoma pathway inactivation dictates response to CDK4/6 inhibition in GBM. P Natl Acad Sci USA.

[15] Brough R, Frankum JR, Sims D, Mackay A, Mendes-Pereira AM, Bajrami I (2011). Functional Viability Profiles of Breast Cancer. Cancer discovery.

[16] Schoolmeesters A, Brown DD, Fedorov Y (2012). Kinome-Wide Functional Genomics Screen Reveals a Novel Mechanism of TNF alpha-Induced Nuclear Accumulation of the HIF-1 alpha Transcription Factor in Cancer Cells. PloS one.

[17] Zha JH, Xia YC, Ye CL, Hu Z, Zhang Q, Xiao H (2021). The Anti-Non-Small Cell Lung Cancer Cell Activity by a mTOR Kinase Inhibitor PQR620. Frontiers in oncology.

[18] Jiao PF, Tang PJ, Chu D, Li YM, Xu WH, Ren GF (2021). Long Non-Coding RNA THOR Depletion Inhibits Human Non-Small Cell Lung Cancer Cell Growth. Frontiers in oncology.

[19] Zha JH, Xia YC, Ye CL, Hu Z, Zhang Q, Xiao H (2021). The Anti-Non-Small Cell Lung Cancer Cell Activity by a mTOR Kinase Inhibitor PQR620. Frontiers in oncology.

[20] Yang H, Zhao M, Zhao L, Li P, Duan Y, Li G (2020). CircRNA BIRC6 promotes non-small cell lung cancer cell progression by sponging microRNA-145. Cellular oncology.

[21] Xue Y, Jiang K, Ou L, Shen M, Yang Y, Lu J (2022). Targeting sphingosine kinase 1/2 by a novel dual inhibitor SKI-349 suppresses non-small cell lung cancer cell growth. Cell death & disease.

[22] Wang Y, Liu YY, Chen MB, Cheng KW, Qi LN, Zhang ZQ (2021). Neuronal-driven glioma growth requires Galphai1 and Galphai3. Theranostics.

[23] Gao YY, Ling ZY, Zhu YR, Shi C, Wang Y, Zhang XY (2021). The histone acetyltransferase HBO1 functions as a novel oncogenic gene in osteosarcoma. Theranostics.

[24] Yin DP, Zheng YF, Sun P, Yao MY, Xie LX, Dou XW (2022). The pro-tumorigenic activity of p38gamma overexpression in nasopharyngeal carcinoma. Cell death & disease.

[25] Bian ZJ, Shan HJ, Zhu YR, Shi C, Chen MB, Huang YM (2022). Identification of Galphai3 as a promising target for osteosarcoma treatment. International journal of biological sciences.

[26] Wu F, Liu F, Dong L, Yang H, He X, Li L (2018). miR-1273g silences MAGEA3/6 to inhibit human colorectal cancer cell growth via activation of AMPK signaling. Cancer letters.

[27] Xu W, Xu L, Chen M, Mao YT, Xie ZG, Wu SL (2012). The effects of low dose X-irradiation on osteoblastic MC3T3-E1 cells in vitro. BMC Musculoskelet Disord.

[28] Xia L, Liu Y, Wang Y (2019). PD-1/PD-L1 Blockade Therapy in Advanced Non-Small-Cell Lung Cancer: Current Status and Future Directions. The oncologist.

[29] Meng X, Liu Y, Zhang J, Teng F, Xing L, Yu J (2017). PD-1/PD-L1 checkpoint blockades in non-small cell lung cancer: New development and challenges. Cancer letters.

[30] Yu H, Chen Y, Jiang P (2018). Circular RNA HIPK3 exerts oncogenic properties through suppression of miR-124 in lung cancer. Biochemical and biophysical research communications.

[31] Zhang B, Lu HY, Xia YH, Jiang AG, Lv YX (2018). Long non-coding RNA EPIC1 promotes human lung cancer cell growth. Biochemical and biophysical research communications.

[32] Jiang M, Qi L, Li L, Li Y (2020). The caspase-3/GSDME signal pathway as a switch between apoptosis and pyroptosis in cancer. Cell death discovery.

[33] Huang K, Fingar DC (2014). Growing knowledge of the mTOR signaling network. Seminars in cell & developmental biology.

[34] Jewell JL, Guan KL (2013). Nutrient signaling to mTOR and cell growth. Trends in biochemical sciences.

[35] Tzatsos A, Tsichlis PN (2007). Energy depletion inhibits phosphatidylinositol 3-kinase/Akt signaling and induces apoptosis via AMP-activated protein kinase-dependent phosphorylation of IRS-1 at Ser-794. The Journal of biological chemistry.

[36] Tan AC (2020). Targeting the PI3K/Akt/mTOR pathway in non-small cell lung cancer (NSCLC). Thoracic cancer.

[37] Vestergaard HH, Christensen MR, Lassen UN (2018). A systematic review of targeted agents for non-small cell lung cancer. Acta oncologica.

[38] Heavey S, O'Byrne KJ, Gately K (2014). Strategies for co-targeting the PI3K/AKT/mTOR pathway in NSCLC. Cancer treatment reviews.

[39] Fumarola C, Bonelli MA, Petronini PG, Alfieri RR (2014). Targeting PI3K/AKT/mTOR pathway in non small cell lung cancer. Biochemical pharmacology.

[40] Skoulidis F, Heymach JV (2019). Co-occurring genomic alterations in non-small-cell lung cancer biology and therapy. Nature reviews Cancer.

[41] Gridelli C, Rossi A, Carbone DP, Guarize J, Karachaliou N, Mok T (2015). Non-small-cell lung cancer. Nature reviews Disease primers.

[42] Herbst RS, Morgensztern D, Boshoff C (2018). The biology and management of non-small cell lung cancer. Nature.

[43] Huang CY, Ju DT, Chang CF, Muralidhar Reddy P, Velmurugan BK (2017). A review on the effects of current chemotherapy drugs and natural agents in treating non-small cell lung cancer. BioMedicine.

[44] Lim M, Park J, Lowe AC, Jeong HO, Lee S, Park HC (2020). A lab-on-a-disc platform enables serial monitoring of individual CTCs associated with tumor progression during EGFR-targeted therapy for patients with NSCLC. Theranostics.

[45] Sohoni S, Ghosh P, Wang TY, Kalainayakan SP, Vidal C, Dey S (2019). Elevated Heme Synthesis and Uptake Underpin Intensified Oxidative Metabolism and Tumorigenic Functions in Non-Small Cell Lung Cancer Cells. Cancer research.

[46] Alam MM, Sohoni S, Kalainayakan SP, Garrossian M, Zhang L (2016). Cyclopamine tartrate, an inhibitor of Hedgehog signaling, strongly interferes with mitochondrial function and suppresses aerobic respiration in lung cancer cells. BMC cancer.

