# Supplementary figures and images for: Identification of the mitochondrial protein ADCK2 as a therapeutic oncotarget of NSCLC
Source: Int J Biol Sci. 2022 Oct 24;18(16):6163–75. doi: 10.7150/ijbs.78354 (PMC9682539; doi:10.7150/ijbs.78354)

**Figure 8**

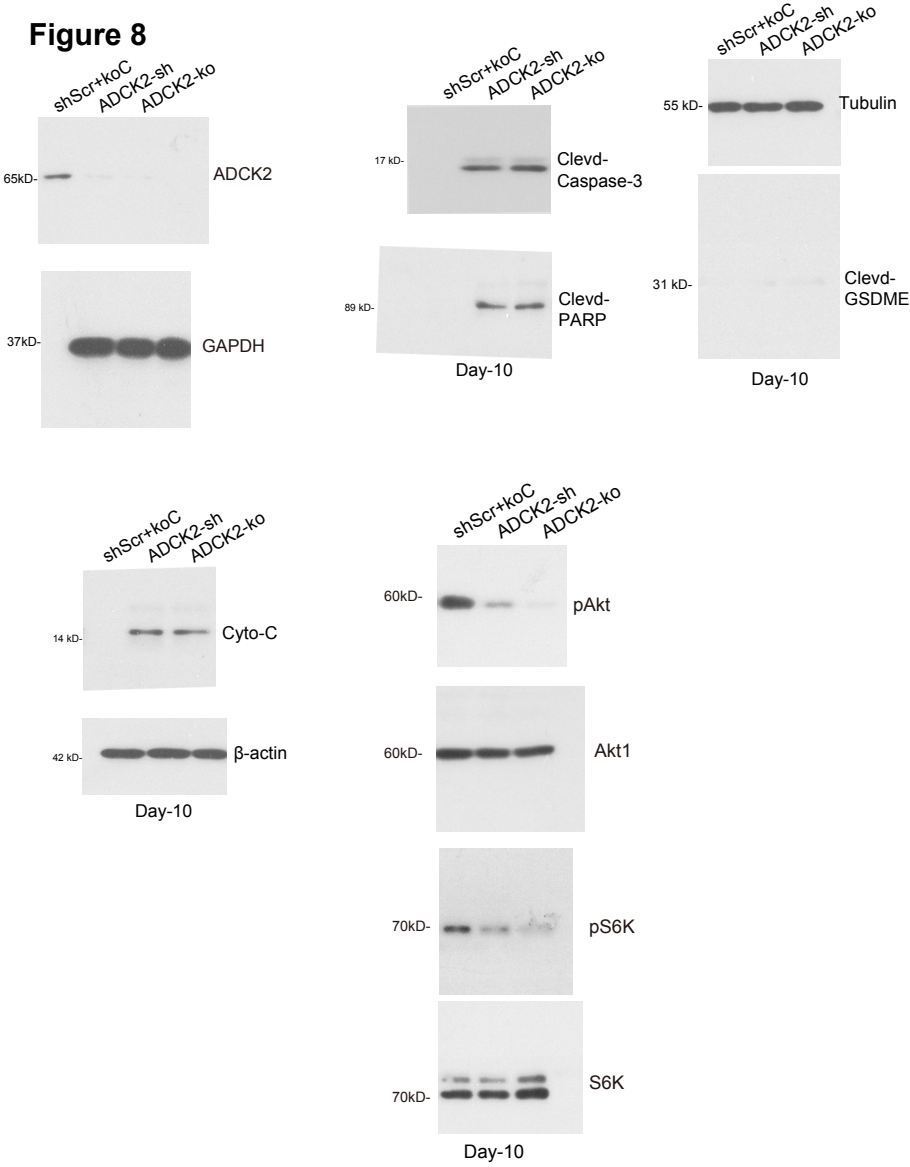

Supplement: Supplementary file 1 — Supplementary figure. [file ijbsv18p6163s1.pdf]
